# Investigation of *Burkholderia cepacia* complex bacteremia outbreak in a neonatal intensive care unit: a case series 

**DOI:** 10.1186/s13256-020-02415-8

**Published:** 2020-06-23

**Authors:** Tanisha Bharara, Anita Chakravarti, Mukesh Sharma, Priti Agarwal

**Affiliations:** Department of Microbiology, SGT University, Gurugram, Haryana India

**Keywords:** Blood culture, Surveillance, Hospital infection control, Multidrug resistance

## Abstract

**Introduction:**

*Burkholderia cepacia* complex is a ubiquitous organism with a high virulence potential. It is found most commonly in moist environments. Hospital outbreaks have been reported from diverse sources such as contaminated faucets, nebulizers, disinfectant solutions, multidose antibiotic vials, tap water, bottled water, nasal sprays, and ultrasound gels. In this article, we present our experience in investigating and successfully managing an outbreak of nosocomial transmission of *Burkholderia cepacia* sepsis in the neonatal intensive care unit at SGT Hospital, Haryana, India.

**Case presentation:**

During the month of March, multiple *Burkholderia cepacia* complex isolates were recovered from blood cultures of Caucasian babies admitted to the neonatal intensive care unit of our hospital. The organisms were multidrug-resistant, with *in vitro* sensitivity to meropenem alone (minimum inhibitory concentration = 4 μg/ml). An outbreak was suspected, and the neonatal intensive care unit in-charge and hospital infection control teams were alerted. Outbreak investigation was initiated, and surveillance samples were collected. *Burkholderia cepacia* complex was successfully isolated from suction apparatus. The isolates were phenotypically typed (biotyping and antimicrobial susceptibility testing) and found to be identical.

**Conclusions:**

In our study, the index case might have been exposed to infection due to a physiological state of low immunity (preterm, low birth weight, and mechanical ventilation). The rest of the cases might have been exposed to this organism due to inadequate hand hygiene/improper cleaning and disinfection practices. Timely reporting and implementation of infection control measures played a significant role in curtailing this outbreak.

## Introduction

*Burkholderia cepacia* complex (BCC) consists of a cluster of 18 closely related genomovars. It has been recognized as an opportunistic human pathogen since the early 1980s [1]. BCC has been isolated from numerous water sources and wet surfaces, including disinfectants and intravenous fluids [2]. Hospital outbreaks have been reported due to anesthetics, disinfectants, intravenous solutions, nebulizer solutions, mouthwash, and medical devices, including respiratory therapy equipment (Table 1).

**Table 1:** Summary of various outbreaks of *Burkholderia cepacia* complex in hospital setup in India over the last 5 years [2–8]

Serial number	Author, location (year)	Place	Number of cases	Duration of outbreak	Site of infection	Source of infection	Mortality rate (%)
1.	Rastogi, New Delhi (2019) [2]	Neurotrauma intensive care unit	48	4 months	Blood and respiratory samples	Water	18.7
2.	Baul, Kolkata (2014) [3]	Hemato-oncology unit	29	6	Blood	Intravenous antibiotics	3.4
3.	Singhal, Mumbai (2015) [4]	Chemotherapy daycare unit	13	Not specified	Blood	Antiemetic drug	Nil
4.	Gupta, Rajastha (2018) [5]	Oncology care center	14	1 month	Blood	Could not be identified	Nil
5.	Mali, Mumbai (2017) [6]	PICU, pediatric ward	76	8 months	Blood	Amikacin vial rubber stopper	Not specified
6.	Antony, Karnataka (2016) [7]	PICU	3	Sporadic episode	Blood	Water	Nil
7.	Yamunadevi, Chennai (2018) [8]	CCU	24	3 months	Blood	Ultrasound gel	Not specified
8.	Present case, Haryana (2019)	NICU	4	1 month	Blood	Suction apparatus	0.53

*PICU* Pediatric intensive care unit, *CCU* Cardiac care unit, *NICU* Neonatal intensive care unit

In this article, we present our experience in investigating and managing an outbreak of nosocomial transmission of *Burkholderia cepacia* sepsis in the neonatal intensive care unit (NICU) at SGT Hospital, Haryana, India.

## Case presentation

During the month of March, three blood cultures received in our microbiology laboratory grew organisms, which were morphologically similar. On further investigation, all the samples were traced to neonates admitted to our NICU. An outbreak was suspected, so the NICU in-charge and hospital infection control (HIC) teams were alerted. A fourth sample was received, with similar growth observed. All patient details were anonymized, coded by randomization, and delinked from any identity of the patients (Table 2).

**Table 2:** Details of cases of *Burkholderia cepacia* complex bloodstream infections in our neonatal intensive care unit (March–April 2019)

Neonate	Term/preterm (weeks)	Birth weight (kg)	Place and mode of delivery	Respiratory support	CRP	Serum procalcitonin	Outcome
**1**	Preterm (32 + 4)	LBW (1.78)	In-born, NVD	CPAP	Positive	Not done	Discharged
**2**	Preterm (29 + 2)	ELBW (0.9)	In-born, NVD	Mechanical ventilation	Not done	Not done	Expired
**3**	Preterm (30 + 6)	LBW (1.64)	In-born, NVD	Mechanical ventilation	Positive	Positive (2.47)	Discharged
**4**	Term (36 + 4)	LBW (1.68)	In-born, NVD	CPAP	Positive	Not done	LAMA

Outbreak investigation was initiated and surveillance samples collected *CRP* C-reactive protein, *LBW* Low birth weight, *NVD* Normal vaginal delivery, *CPAP* Continuous Positive Airway Pressure, *ELBW* Extremely low birth weight infant, *LAMA* Leave against medical advice

### Patient 1 (32 + 4 weeks of gestation)

A preterm (32 + 4 weeks of gestation), low-birth-weight (1.78 kg) Caucasian male baby was delivered by normal vaginal route at our hospital. The mother had preterm rupture of membrane since 20 days and was receiving antibiotics. The baby cried immediately after birth; however, subsequently, the baby showed signs of respiratory distress (nasal flaring, chest retractions, respiratory rate of 48 breaths/minute). The baby was treated with continuous positive airway pressure (CPAP). Routine investigations showed leukocytosis and metabolic acidosis. The baby’s C-reactive protein (CRP) became positive after 48 hours. A blood culture was sent, and the baby was started on intravenous injections of cefotaxime (90 mg in 10 ml of normal saline twice per day) and amikacin (32 mg every 36 hours). Subsequently, a blood culture grew BCC, and antibiotic treatment was changed to injectable meropenem. The patient improved and was discharged.

### Patient 2 (29 + 2 weeks of gestation)

A preterm (29 + 2 weeks of gestation), extremely low-birth-weight (900 g) Caucasian male baby was delivered by a primigravida by normal vaginal delivery at SGT Hospital. The mother reported a history of oligohydramnios with premature rupture of the membranes since 12 days. The baby was admitted to the NICU of our hospital on day 1 of life. The baby did not cry immediately after birth and was admitted with the complaints of metabolic acidosis, seizures, and shock with sepsis. On day 2 of life, the baby was shifted to mechanical ventilation. A blood culture was sent on day 1 of admission. Empirical treatment with intravenous injections of cefotaxime (90 mg in 10 ml of normal saline twice per day) and amikacin (32 mg every 36 hours) was started. Subsequently, a blood culture grew BCC (day 2), and antibiotic treatment was changed to injectable meropenem. However, the baby did not survive and was declared dead on day 6.

### Patient 3 (30 + 6 weeks of gestation)

A preterm (30 + 6 weeks of gestation), 1.64-kg Caucasian female baby born to a primigravida by normal vaginal delivery was admitted to the NICU of our hospital on day 1 of life. The mother reported a history of preeclampsia during the antenatal period. The baby presented with severe anemia, generalized edema, and pansystolic murmur. She subsequently developed respiratory distress and was put on mechanical ventilation. A blood culture was positive for BCC on day 15.

### Patient 4 (32 + 4 weeks of gestation)

Patient 4 was a term (32 + 4 weeks of gestation), small for gestation age, low-birth-weight (1.68 kg) Caucasian male baby born to a G2 P1 L1 A0 female. The baby was delivered by normal vaginal delivery at SGT Hospital. The baby cried after 1 minute of bag and mask ventilation, a case of perinatal asphyxia. He was admitted to the NICU of our hospital on day 1 of life and subsequently started on CPAP therapy. A blood culture was sent on day 2 of admission. Routine blood tests showed normocytic, normochromic anemia and leukopenia. The patient’s CRP test result was positive. His blood culture result was positive on day 3 of life. He left under medical advice on day 4.

### Microbiological analysis

#### Blood culture

All the blood culture samples were collected in BacT/ALERT aerobic blood culture bottles (bioMérieux, New Delhi, India) and sent to the hospital’s microbiology laboratory. The samples were incubated and monitored regularly using the BacT/ALERT system (bioMérieux). All bottles with positive signals were removed from the instrument, Gram-stained, and subcultured on blood agar and MacConkey agar plates. On blood agar, the organism grew as opaque, glistening colonies, nonpigmented initially, later developing yellowish pigmentation and non-lactose-fermenting colonies on MacConkey agar. It was catalase-positive and oxidase-positive and produced acid from glucose, mannitol, lactose, and sucrose oxidatively. The isolates decarboxylated lysine and ornithine and were resistant to polymyxin B and colistin. The organism was presumed to be BCC, and the finding was confirmed with the help of VITEK 2 (bioMérieux). Antimicrobial susceptibility was determined by both the Kirby-Bauer disc diffusion method in accordance with the Clinical and Laboratory Standards Institute (2019) recommendations and the VITEK 2 antimicrobial susceptibility testing (AST) card (bioMérieux) [9]. The organism was sensitive only to meropenem (4 μg/ml). It was resistant to piperacillin/tazobactam (> 128 μg/ml), cefoperazone/sulbactam (16 μg/ml), cefepime (4 μg/ml), imipenem (> 16 μg/ml), amikacin (> 64 μg/ml), gentamicin (> 16 μg/ml), ciprofloxacin (2 μg/ml), tigecycline (> 8 μg/ml), colistin (> 16 μg/ml), and trimethoprim/sulbactam (160 μg/ml).

#### Surveillance cultures

NICU surveillance samples were collected with the help of sterile swabs and sent immediately to our hospital’s microbiology laboratory. Samples were taken from ventilator tubes, suction apparatus, Ambu bags (Ambu, Ballerup, Denmark), Cheatle forceps, injection preparation areas, amikacin vials, taps, bed rails, and sterile saline for injection preparations. The swabs were plated on blood agar plates and incubated overnight at 36 ± 1 °C under aerobic conditions. The plates were read the next day, and colonies were identified with the help of Gram staining and biochemical tests. The isolates were confirmed using the VITEK 2 system (bioMérieux). Antimicrobial susceptibility of the clinical isolates was determined by both the Kirby-Bauer disc diffusion method and the VITEK 2 AST card. BCC was isolated from a surveillance sample of a suction bottle. All five isolates (clinical = 4 and surveillance = 1) were phenotypically typed (AST) and found to be identical.

Cohorting of cases was done. Treatment of babies was changed to injection meropenem. Suction bottles were cleaned with thorough scrubbing followed by decontamination with 2% glutaraldehyde solution. Retraining on hand hygiene, cleaning, and disinfection procedures was provided. The organism was not isolated again. The mortality rate for this outbreak was found to be 0.53%.

## Discussion and conclusions

Healthcare-associated infections are defined as infections that were neither present nor incubating at the time a patient was admitted to a healthcare facility [10]. According to our infection control policy, an outbreak is suspected when an infection is isolated from two or more patients in a defined time frame. For our study, an outbreak was defined as simultaneous presence of more than two patients with positive culture results for BCC.

Outbreak cases were defined as a neonate with a clinical suspicion of sepsis (fever, tachycardia, tachypnea, leukocytosis, or leukopenia, with or without hypotension) who had one or more BCC-positive blood culture results. The outbreak period was defined as the time between March 2019 and April 2019.

We have a very vigilant hospital infection control team. Our infection control officer takes regular rounds along with our infection control nurses. The cluster of BCC infections was observed in our nine-bed NICU. The nurse-to-patient ratio and the doctor-to-patient ratio in the NICU are usually 1:4 and 1:3, respectively. The outbreak was suspected in March 2019, and an investigation was triggered when four subsequent cases of bacteremia caused by *B. cepacia* occurred over a period of 1 month. This prompted a detailed microbiological investigation and hospital infection surveillance activities.

BCC is an opportunistic pathogen of high virulence potential. Various virulence mechanisms associated with this organism are multidrug resistance (bcrA efflux pump), genes determining transmissibility (*esmR* and *cblA* genes, and *esmR*), siderophores (salicylic acid, ornibactin, pyochelin, and cepabactin), and adherence proteins (long flexible type II pili) [11]. They have the potential to survive and multiply in the presence of disinfectants, indwelling invasive medical devices, and antibiotic solutions, thus acting as a potential reservoir for infections in the hospital setting [12, 13].

Minimum inhibitory concentration (MIC) determination of our isolates revealed multidrug resistance. Our isolate showed *in vitro* resistance to trimethoprim-sulfamethoxazole (TMP-SMZ), with an MIC of 160. Rates of *in vitro* resistance of BCC to TMP-SMZ range from 5% in Quebec, Canada, and Latin America to 10% in Europe [14, 15]. According to the Clinical and Laboratory Standards Institute guidelines, the antibiotics effective against BCC include levofloxacin, meropenem, cotrimoxazole, ceftazidime, and minocycline [9]. Though BCC organisms are highly resistant, antibiotic combinations have been found to be effective in a few studies [16, 17].

In our study, all the isolates from patients and the environmental samples belonged to the same biotype and exhibited the same antibiogram where the isolate was sensitive to meropenem alone. The index case might have acquired the infection due to a physiological state of low immunity (preterm, low birth weight, and mechanical ventilation). The rest of the cases might have been exposed to this organism due to inadequate hand hygiene practices/improper cleaning and disinfection practices. In a study by Mali *et al.* [6], BCC was isolated from the upper surface of the rubber stopper of sealed multidose amikacin injection vials. It was hypothesized that the needle might have become contaminated while amikacin solution was aspirated from the vials [6]. As per our hospital antibiotic policy, all these babies were started on empirical treatment with intravenous injections of cefotaxime and amikacin while blood culture results were awaited. This might have been another risk factor in the spread of BCC sepsis, since the organism was resistant to these antimicrobials.

The efficacy of control measures was evaluated by continued follow-up of cases after the outbreak, both clinically and microbiologically. Control measures were considered effective because new cases of BCC sepsis ceased to occur. Timely reporting to the clinician, implementation of infection control measures such as hand hygiene, proper cleaning, and disinfection of NICU equipment, and cohorting of infected cases curtailed this outbreak.

## Data Availability

The datasets used and/or analyzed during the current study are available from the corresponding author on reasonable request.

## Abbreviations

BCC: *Burkholderia cepacia* complex
CPAP: Continuous positive airway pressure
CRP: C-reactive protein
HIC: Hospital infection control
LBW: Low birth weight
NICU: Neonatal intensive care unit
